# Primary tumor size as a prognostic factor in duodenal neuroendocrine neoplasms ≤20 mm: a population-based SEER analysis

**DOI:** 10.1530/EO-25-0108

**Published:** 2026-02-11

**Authors:** Giuseppe Lamberti, Francesco Panzuto, Maria Rinzivillo, Flaminia Benedetta Zoboli, Irene Zannini, Elisa Andrini, Elisabetta Dell’Unto, Riccardo Di Pangrazio, Davide Campana

**Affiliations:** ^1^ Department of Medical and Surgical Sciences (DIMEC), Alma Mater Studiorum – University of Bologna, Bologna, Italy; ^2^ Department of Medical-Surgical Sciences and Translational Medicine, Sapienza Università di Roma, Digestive Disease Unit, ENETS Center of Excellence, Sant’Andrea University Hospital, Rome, Italy

**Keywords:** duodenal neuroendocrine neoplasms, tumor size, SEER, lymph-node metastasis, endoscopic resection

## Abstract

**Objective:**

The prognostic value of primary tumor size in duodenal neuroendocrine neoplasms (dNENs) ≤20 mm remains uncertain. Although size is used to guide management, its independent effect on survival is unclear.

**Design and methods:**

We performed a population-based study using the US SEER database (1975–2021), including adults with histologically confirmed dNENs ≤20 mm. Disease-specific survival (DSS) was the primary endpoint. Associations between tumor size, stage, lymph-node involvement, and DSS were evaluated using chi-squared tests, logistic regression, Kaplan–Meier curves, and Cox models. Receiver operating characteristic (ROC) analysis identified the optimal size cutoff. Mediation analysis assessed whether stage or grade mediated the size–DSS relationship.

**Results:**

Among 3,515 eligible cases, median age was 65 years, 51% were male, and 89% had well-differentiated NETs. Most tumors were localized (85.2%) and ≤10 mm (74.4%). Median follow-up was 56 months, with 160 disease-specific deaths (4.6%). In univariable analysis, size >10 mm was associated with worse DSS, but the association disappeared after adjustment for grade, stage, and surgery. Grade G3 and advanced stage independently predicted poorer survival, while radical surgery was protective. Larger tumors were more frequently high grade and advanced stage (*P* < 0.001), and each 1 mm increase raised the odds of nodal metastasis by 12% (OR 1.12). ROC analysis identified a size threshold of 10.5 mm (AUC = 0.74). Stage mediated only ∼2% of the size–DSS association.

**Conclusions:**

In duodenal NENs ≤ 20 mm, tumor size >10 mm correlates with disease extent but does not independently affect DSS, supporting its role as a stratification marker rather than a prognostic factor.

## Introduction

Neuroendocrine neoplasms (NENs) are rare malignancies whose incidence has markedly increased in recent decades. The most recent SEER-based analysis including over 145,000 US cases (1975–2021) reported a 5.2-fold rise in age-adjusted incidence – from 1.6 to 8.5 per 100,000 persons – and a 20-year prevalence approaching 244,000 cases in 2021 (1). This rise was driven mainly by early-stage and well-differentiated tumors, with a median overall survival exceeding 30 years for localized disease, confirming their generally indolent behavior.

Duodenal neuroendocrine neoplasms (dNENs) account for approximately 2–3% of gastroenteropancreatic NENs. They are usually small, well-differentiated, and incidentally discovered, but some show regional or distant spread, particularly when larger than 10 mm or poorly differentiated. Current ENETS guidelines adopt a 10 mm cutoff to distinguish candidates for endoscopic versus surgical resection, using tumor size as a surrogate of malignant potential (2, 3).

However, whether size independently predicts survival remains uncertain. Several series have linked larger lesions to lymph-node metastases (4, 5, 6, 7), whereas population-based analyses found that the effect of size disappeared after adjustment for grade and stage (8, 9, 10). These discrepancies suggest that size mirrors disease extent rather than acting as an autonomous prognostic factor.

This study aimed to clarify the prognostic and clinicopathologic role of tumor size in duodenal NENs ≤ 20 mm using SEER data, assessing i) its association with disease-specific survival (DSS), ii) its correlation with stage and lymph-node involvement, iii) the optimal dimensional cutoff, and iv) the mediation effect of grade or stage on survival.

## Materials and methods

This retrospective, population-based cohort study was conducted using data from the Surveillance, Epidemiology, and End Results (SEER) program of the US National Cancer Institute. Data from three registry groupings (SEER 8, SEER 12, and SEER 17) covering cases diagnosed between 1975 and 2021 were harmonized into a single analytic dataset according to SEER data-use policies. The registries collectively capture approximately 35% of the US population and include standardized demographic, clinicopathologic, treatment, and survival information. The study followed the Strengthening the Reporting of Observational Studies in Epidemiology (STROBE) guidelines.

### Study population

All cases of duodenal and ampullary neuroendocrine neoplasms (NENs) recorded in SEER between 1975 and 2021 were screened. Eligible cases were identified using ICD-O-3 topography codes C17.0 (duodenum) and C24.1 (ampulla of Vater) combined with morphology codes 8240/3, 8241/3, 8249/3, and 8246/3. Inclusion criteria were as follows: i) age ≥ 18 years at diagnosis, ii) histologically confirmed NENs, iii) available information on primary tumor size (≤20 mm for the main analytic cohort), and iv) known DSS status. Exclusion criteria were as follows: i) missing or inconsistent data for key variables (size, histology, or survival), ii) secondary or metastatic tumors, and iii) duplicate or overlapping entries identified via SEER unique identifiers.

### Variables and endpoints

Extracted variables included age, sex, race, primary site (duodenum or ampulla), histologic subtype, grade, stage, type of surgery, tumor size, and lymph-node status. In SEER, tumors coded as ‘carcinoid tumors’ were classified as neuroendocrine tumors (NETs), and this terminology was retained. Tumor grade was extracted from the SEER database using the standard SEER grading variable, which reflects the degree of histologic differentiation of the tumor. According to SEER coding definitions, grade 1 corresponds to well-differentiated tumors, grade 2 to moderately differentiated tumors, grade 3 to poorly differentiated tumors, and grade 4 to undifferentiated or anaplastic tumors. For analytic purposes, grades 3 and 4 were combined into a single high-grade category (G3), given their shared representation of poorly differentiated or undifferentiated disease and their similar adverse prognostic implications. Cases with missing or unspecified grade were retained as a separate category and were not imputed.

Tumor size was collected as a continuous variable (mm) and dichotomized as ≤10 mm versus >10 mm. In the SEER database, tumor size reflects the best available measurement recorded at diagnosis, derived from pathology, endoscopic assessment, imaging studies, or a combination thereof; the specific source of measurement cannot be systematically ascertained. Lymph-node involvement was defined as ≥1 positive node among those examined. Surgical treatment was categorized in SEER as radical surgery, local resection, no surgery, or surgery, not otherwise specified. The ‘no surgery’ category indicates the absence of a recorded surgical procedure on the primary tumor and does not necessarily imply active surveillance. The ‘unspecified’ category reflects cases in which a procedure was performed, but details on the type of intervention were not available; this group may include endoscopic or limited surgical procedures.

The primary endpoint was DSS, defined as the time in months from diagnosis to death attributed to the NEN, as reported in the SEER cause-specific death classification. Patients alive or deceased from other causes were censored. Secondary endpoints included associations between tumor size and stage or lymph-node status, determination of the optimal size cutoff for advanced disease, and mediation of the size–DSS relationship by stage or grade.

### Data quality and ethics

Data consistency was verified through automated validation procedures. Records with missing or implausible values were excluded by list-wise deletion (complete-case analysis). No imputation was performed. The study was conducted in accordance with the Declaration of Helsinki and its later amendments. Because it used de-identified, publicly available SEER data, formal institutional review board approval and informed consent were not required.

### Statistical analysis

All analyses were performed using R software (version 4.3.2; R Foundation for Statistical Computing, Vienna, Austria) with the packages tidyverse 2.0.0, survival 3.5–7, survminer 0.4.9, broom 1.0.5, rstatix 0.7.2, pROC 1.18.4, and mediation 4.5.0.

Continuous variables were summarized as mean ± standard deviation, median, and interquartile range (IQR); categorical variables as counts and percentages. Between-group comparisons were performed using chi-squared or Fisher’s exact test for categorical variables (Cramer’s V for effect size) and Student’s *t*-test or Wilcoxon rank-sum test for continuous variables after Shapiro–Wilk normality assessment.

DSS was analyzed using Kaplan–Meier estimates and compared by log-rank test. Cox proportional-hazards models were fitted using the observed time in months from diagnosis to last follow-up or death and used to estimate hazard ratios (HRs) and 95% confidence intervals (CIs). The proportional-hazards assumption was verified using Schoenfeld residuals (cox.zph function). Variables with *P* < 0.10 in univariable analysis or of clinical relevance were entered into the multivariable model. Associations between tumor size and prognostic variables were assessed using chi-squared or Fisher’s exact test, or logistic regression as appropriate. Receiver operating characteristic (ROC) curves were used to identify the optimal size cutoff (Youden index). Mediation analysis evaluated the indirect effect of stage or grade on the size–DSS relationship using a nonparametric bootstrap (1,000 resamples).

## Results

### Baseline characteristics and follow-up

**Figure 1:**
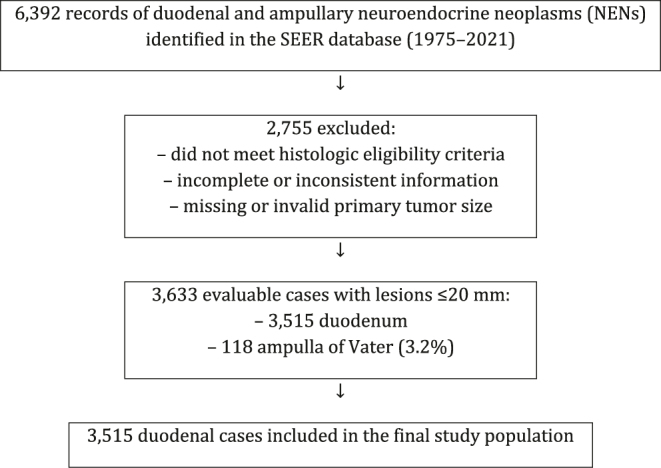
Flowchart of case selection from the SEER database. Flow diagram showing the selection of patients with duodenal neuroendocrine neoplasms ≤20 mm from the SEER 1975–2021 dataset. After exclusion of 2,755 ineligible or incomplete cases, 3,633 evaluable lesions ≤20 mm were identified (3,515 duodenum; 118 ampulla of Vater). Analyses were restricted to the 3,515 duodenal cases.

From an initial cohort of 6,392 records of duodenal and ampullary neuroendocrine neoplasms (NENs) identified in the SEER database (1975–2021), 2,755 cases were excluded because they did not meet the histologic eligibility criteria, had incomplete or inconsistent information required for the analysis, or lacked valid data on primary tumor size. Among the 3,633 evaluable cases with lesions ≤20 mm, 3,515 (96.8%) originated in the duodenum and 118 (3.2%) in the ampulla of Vater. Analyses were restricted to the 3,515 duodenal cases, which represented the final study population (Fig. 1).

The median age at diagnosis was 65 years (IQR 56–73), and 51% were male. The majority of tumors were NETs (89.2%), whereas NECs accounted for 10.8%. Grading was G1 in 61.9%, G2 in 5.6%, G3 in 3.2%, and unknown in 29.3% of cases. Most tumors were localized (85.2%) at presentation, with 7.8% regional, 2.2% distant, and 4.9% unstaged.

Surgical resection was performed in 75% of patients, including radical surgery in 37.7% and local resection in 8.1%, while 21.5% did not undergo surgery and 30.0% had unspecified procedure details (Table 1).

**Table 1:** Baseline characteristics of patients with duodenal neuroendocrine neoplasms ≤20 mm (*n* = 3,515).

Characteristic	*n* (%) or value
Total cases	3,515
Age, years	
Mean ± SD	63.9 ± 12.2
Median (IQR)	65 (56–73)
Range	22–85
Sex	
Male	1,792 (51.0%)
Female	1,723 (49.0%)
Histology	
NET	3,137 (89.2%)
NEC	378 (10.8%)
Grade	
G1	2,176 (61.9%)
G2	196 (5.6%)
G3	113 (3.2%)
Unknown	1,030 (29.3%)
Stage (SEER summary)	
Localized	2,994 (85.2%)
Regional	273 (7.8%)
Distant	76 (2.2%)
Unknown	172 (4.9%)
Surgery type	
Radical	1,325 (37.7%)
Local	284 (8.1%)
None	756 (21.5%)
Unspecified	1,053 (30.0%)
Follow-up (months)	
Observed median (IQR)	53 (21–102)
Range	0–416
Disease-specific deaths	160 (4.6%)

### Disease-specific survival

At a median follow-up of 56 months (95% CI: 54–59) estimated by the reverse Kaplan–Meier method, 160 disease-specific deaths (4.6%) were recorded among 3,515 patients with duodenal NENs ≤ 20 mm.

**Figure 2:**
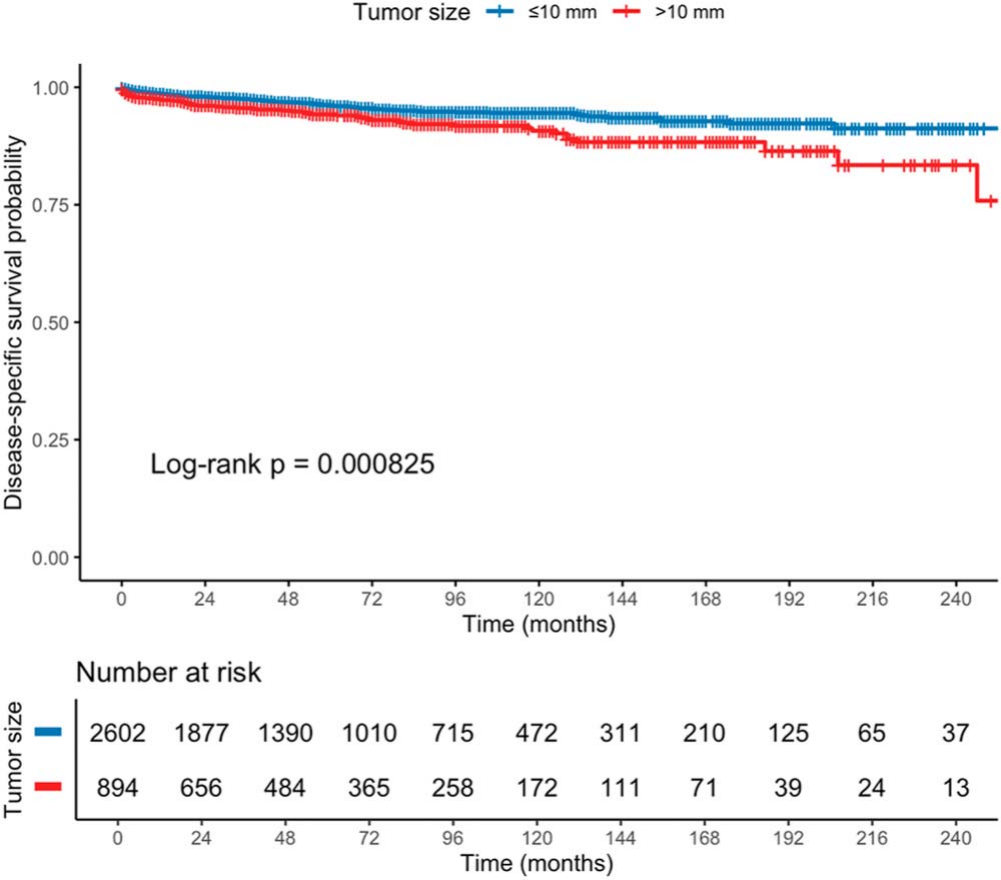
Kaplan–Meier DSS by tumor size Kaplan–Meier curves comparing DSS in patients with duodenal neuroendocrine neoplasms ≤10 mm versus >10 mm. Lesions >10 mm were associated with significantly shorter survival in univariable analysis (log-rank *P* = 0.0008).

In univariable Kaplan–Meier analysis, patients with tumors >10 mm had a significantly shorter DSS compared with those ≤10 mm (log-rank *P* = 0.0008; Fig. 2). The median DSS was not reached in either group, but separation of the survival curves became evident within the first 60 months.

In the univariable Cox analysis (Table 2), tumor size >10 mm was associated with a higher risk of disease-specific death (HR 1.72; 95% CI: 1.25–2.36; *P* = 0.001). Additional adverse prognostic factors included NEC histology (HR 1.74; 95% CI: 1.18–2.58; *P* = 0.006), high-grade disease (G3 vs G1: HR 4.03; 95% CI: 2.00–8.14; *P* < 0.001), and advanced stage (regional HR 2.14; distant HR 7.03; both *P* ≤ 0.002). Increasing age was also independently associated with poorer DSS (HR 1.04 per year; *P* < 0.001), whereas radical surgery markedly reduced disease-specific mortality (HR 0.29; 95% CI: 0.18–0.46; *P* < 0.001).

**Table 2:** Univariable and multivariable Cox proportional-hazards models for DSS in duodenal neuroendocrine neoplasms ≤20 mm (*n* = 3,515).

Variable	Comparison	Univariable	*P*-value	Multivariable	*P*-value
		HR (95%CI)		HR (95%CI)	
Tumor size (>10 vs ≤10 mm)		1.72 (1.25–2.36)	**0.001**	0.88 (0.54–1.45)	0.628
Histology	NEC vs NET	1.74 (1.18–2.58)	**0.006**	1.50 (0.88–2.53)	0.134
Grade	G2 vs G1	1.08 (0.54–2.17)	0.825	0.94 (0.45–1.98)	0.874
	G3 vs G1	4.03 (2.00–8.14)	**<0.001**	3.59 (1.75–7.39)	**<0.001**
Stage	Regional vs localized	2.14 (1.34–3.41)	**0.002**	2.17 (1.11–4.21)	**0.023**
	Distant vs localized	7.03 (4.32–11.46)	**<0.001**	4.49 (2.09–9.67)	**<0.001**
Surgery type	Local vs none	0.67 (0.37–1.20)	0.179	0.39 (0.18–0.87)	**0.022**
	Radical vs none	0.29 (0.18–0.46)	**<0.001**	0.22 (0.11–0.43)	**<0.001**
	Unspecified vs none	0.62 (0.42–0.92)	**0.016**	0.50 (0.29–0.86)	**0.012**
Age (continuous)	Per 1-year increase	1.04 (1.02–1.05)	**<0.001**	1.04 (1.02–1.06)	**<0.001**

Proportional-hazards assumption: global *P* = 0.567.

DSS, disease-specific survival. Bold indicates statistical significance (*P* < 0.05).

In the multivariable Cox model, the prognostic effect of tumor size did not retain independent significance (adjusted HR 0.88; 95% CI: 0.54–1.45; *P* = 0.63). Independent predictors of shorter DSS included grade G3 (HR 3.59; 95% CI: 1.75–7.39; *P* = 0.0005) and advanced stage (regional HR 2.17; distant HR 4.49; both *P* ≤ 0.03), whereas radical surgery remained protective (HR 0.22; 95% CI: 0.11–0.43; *P* < 0.001). The proportional-hazards assumption was satisfied (global test *P* = 0.567).

### Associations of tumor size with disease extent and lymph-node involvement

When stratified by tumor size (≤10 vs >10 mm), several clinicopathologic variables were significantly associated with larger lesions (Table 3). Tumors >10 mm were more frequent in males (*P* = 0.010) and were associated with NEC histology (*P* = 0.002), higher grade (*P* = 0.0001), and advanced stage (*P* < 0.0001; Cramer’s V = 0.27). Surgical treatment also differed by size category (*P* < 0.0001), with larger tumors more often managed with radical surgery. No significant differences were observed in age (*P* = 0.78), the number of lymph nodes examined (*P* = 0.65), or overall survival (*P* = 0.37).

**Table 3:** Associations between dichotomized tumor size (≤10 vs >10 mm) and clinicopathologic features in duodenal neuroendocrine neoplasms ≤20 mm (*n* = 3,515).

Variable	Test	*P*-value	Effect size/statistic
Sex (male vs female)	Chi-squared test	0.010	Cramer’s *V* = 0.044
Race	Chi-squared test	0.724	Cramer’s *V* = 0.014
Histology (NEC vs NET)	Chi-squared test	0.002	Cramer’s *V* = 0.053
Grade	Chi-squared test	0.0001	Cramer’s *V* = 0.085
Stage (localized vs advanced)	Chi-squared test	<0.0001	Cramer’s *V* = 0.273
Surgery type	Chi-squared test	<0.0001	Cramer’s *V* = 0.202
Age (years)	Wilcoxon test	0.784	—
Lymph nodes examined	Wilcoxon test	0.647	—
Positive lymph nodes (median)	Wilcoxon test	<0.0001	—
Overall survival (months)	Wilcoxon test	0.371	—
Correlation: tumor size vs the number of positive nodes	Spearman	<10^−12^	*ρ* = 0.287
Logistic regression: LN + per 1 mm increase	—	<0.001	OR 1.12 (95% CI: 1.08–1.16)

Lymph-node status correlated strongly with tumor size. The median number of positive nodes was 0 in lesions ≤10 mm and 1 in those >10 mm (*P* < 0.0001). The correlation between tumor size and the number of positive nodes was significant (Spearman *ρ* = 0.287, *P* < 10^−12^). Logistic regression confirmed that each 1 mm increase in tumor size raised the odds of nodal positivity by 12% (OR 1.12; 95% CI: 1.08–1.16; *P* < 0.001).

**Figure 3:**
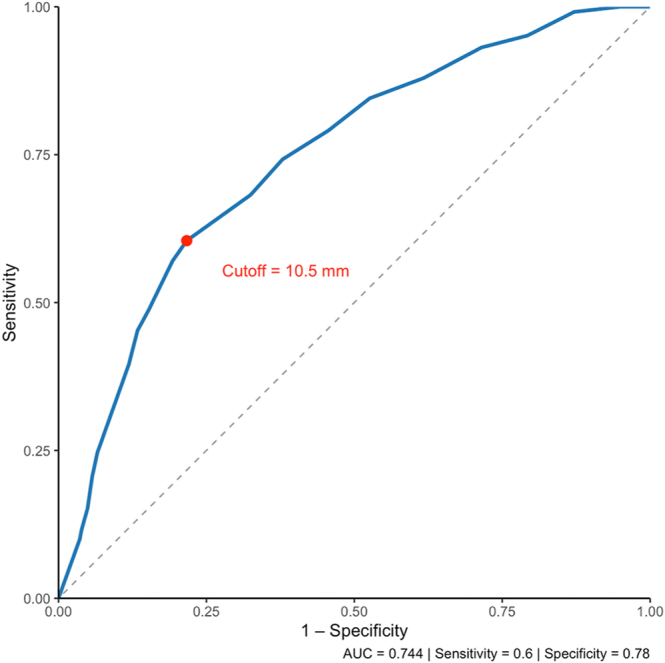
ROC curve for predicting advanced disease based on tumor size. Receiver operating characteristic (ROC) curve evaluating tumor size as a predictor of advanced disease (regional/distant stage). The optimal cutoff identified was 10.5 mm (AUC = 0.744), supporting a clinically relevant threshold near 10 mm.

**Figure 4:**
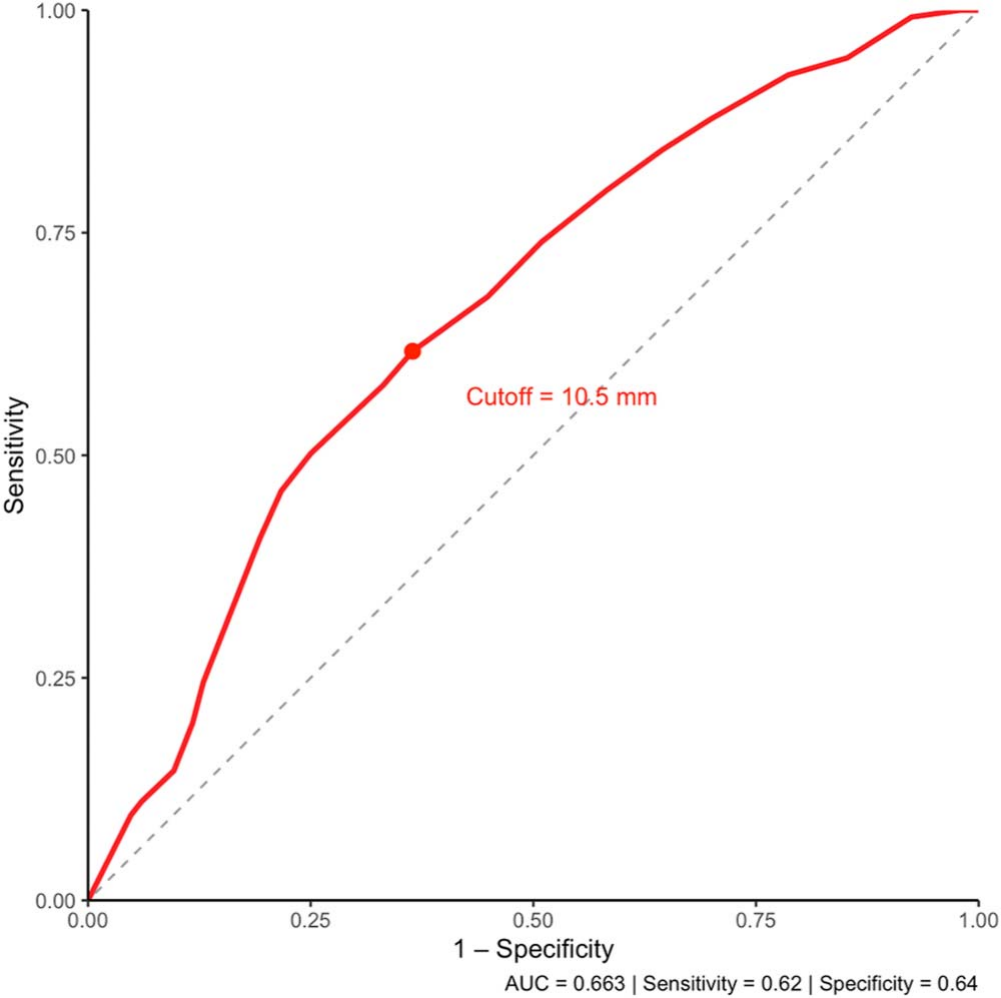
ROC curve for predicting lymph-node metastasis based on tumor size. Receiver operating characteristic (ROC) curve assessing the discriminative accuracy of tumor size for lymph-node positivity. The optimal cutoff was 10.5 mm (AUC = 0.663), indicating moderate predictive ability and alignment with the 10 mm threshold.

Receiver operating characteristic (ROC) analysis identified a cutoff of 10.5 mm as the optimal threshold to discriminate between localized and advanced disease (AUC = 0.744; sensitivity 0.60; specificity 0.78) and between node-negative and node-positive cases (AUC = 0.663; sensitivity 0.62; specificity 0.64). Both analyses supported a dimensional threshold near 10 mm as a clinically relevant boundary for risk stratification (Figs 3 and 4).

It should be acknowledged that the predominance of tumors ≤10 mm in this cohort may affect the stability of ROC analysis-derived thresholds. Accordingly, the 10.5 mm cutoff identified by ROC analysis should be interpreted as a supportive finding rather than a definitive discriminative boundary, reinforcing – rather than redefining – the clinically adopted 10 mm threshold. To determine whether these associations were independent of tumor grade, multivariable logistic regression models were fitted including both tumor size (>10 vs ≤10 mm) and grade as covariates. Tumor size >10 mm remained strongly associated with advanced stage (OR 5.37; 95% CI: 4.13–7.01; *P* < 0.001) and lymph-node positivity (OR 2.27; 95% CI: 1.56–3.31; *P* < 0.001), confirming that these correlations were independent of grade.

### Mediation analysis

To evaluate whether the association between tumor size and DSS was mediated by disease stage or grade, two separate causal mediation models were fitted (Table 4). When stage was included as a mediator, the average causal mediation effect (ACME) was small but statistically significant (ACME = 0.0069; *P* = 0.002), while the average direct effect (ADE) was not (ADE = −0.0031; *P* = 0.66). Approximately 2.0% of the total effect of tumor size on DSS was mediated through stage.

**Table 4:** Mediation analysis of the association between tumor size (>10 vs ≤10 mm) and 5-year DSS in duodenal NENs ≤ 20 mm.

Mediator	ACME	*P*-value	ADE	*P*-value	% Mediated
Stage	0.0069	0.002	−0.0031	0.66	2.0%
Grade	0.00047	0.116	−0.0036	0.60	−0.7%

DSS, disease-specific survival.

When grade was tested as a mediator, neither the indirect (ACME = 0.00047; *P* = 0.116) nor direct effect (ADE = −0.0036; *P* = 0.60) reached statistical significance, and the proportion mediated was negligible (−0.7%).

Overall, these findings indicate that tumor size was not an independent prognostic factor for DSS but was strongly and independently associated with disease extent and nodal involvement, with minimal mediation by grade or stage.

## Discussion

In this population-based cohort of duodenal neuroendocrine neoplasms (dNENs) ≤20 mm, tumor size >10 mm was associated with worse DSS in univariate analysis, but this effect disappeared after adjustment for grade, stage, and surgery. Grade G3 and advanced stage remained independent adverse factors, while radical surgery was protective, indicating that tumor size reflects disease extent and biologic aggressiveness rather than acting as an autonomous prognostic variable.

### Primary endpoint: disease-specific survival

The apparent prognostic impact of tumor size was fully explained by its correlation with grade and stage. Once these covariates were considered, the independent contribution of size to DSS was null, confirming that outcome in small duodenal NENs is primarily driven by tumor grade, as captured by SEER grading, and disease extent. It should be noted that SEER grade may encompass both well-differentiated and poorly differentiated high-grade neoplasms, which represents an inherent limitation of registry-based analyses.

These results mirror those of Randle *et al.*, who found that size lost significance after stage adjustment in SEER data, and of Untch *et al.*, where recurrence risk was determined by invasion depth and grade rather than diameter (8, 9). Similarly, Vanoli *et al.* highlighted that morphologic pattern and proliferative index outweigh dimensional criteria in predicting prognosis (10). Collectively, these studies and our findings indicate that tumor size acts as a surrogate marker of tumor biology and dissemination, not as an independent driver of survival (11, 12).

This clarification supports a shift from rigid dimensional thresholds to integrated prognostic models incorporating grade, stage, and completeness of resection. Such models more accurately reflect the biological heterogeneity of dNENs and may prevent overtreatment of small, low-grade localized lesions.

### Secondary endpoints: stage and lymph-node involvement

A strong quantitative association was observed between tumor size and disease extent. When stratified by tumor size (≤10 vs >10 mm), several clinicopathologic variables were significantly associated with larger lesions (Table 3). Tumors > 10 mm were more frequent in males; however, the strength of this association was weak (Cramer’s *V* = 0.044), and sex did not independently influence DSS, suggesting limited clinical relevance. Lesions exceeding 10 mm were significantly more likely to present with regional or distant spread and lymph-node metastases, independent of grade. ROC analysis consistently identified a threshold around 10 mm for both parameters.

These data align with Hatta *et al.* and Fujii *et al.*, who reported similar cutoffs and comparable discriminative accuracy in resected series, and confirm previous SEER-based analyses, indicating that small duodenal NETs rarely show nodal involvement below this threshold (5, 13, 14, 15). Findings from Zhang *et al.* and Dogeas *et al.* further support the predictive value of tumor size for nodal spread despite its lack of independent prognostic weight (6, 7).

Taken together, these results validate the 10 mm cutoff as a practical tool for estimating the probability of nodal or regional disease. Size retains clinical and statistical relevance as a stratification factor that can guide preoperative staging and the extent of surgical intervention, even if it does not directly determine long-term survival (2, 16, 17).

### Integration and clinical implications

The convergence of evidence from this study and prior literature clarifies the dual role of tumor size in dNENs. While not a prognostic determinant of DSS, it remains a reliable marker of disease extent. By analyzing the largest population cohort restricted to lesions ≤20 mm and applying mediation modeling, this study quantitatively defines that relationship and provides robust, population-based confirmation of the 10 mm operational threshold endorsed by ENETS guidelines (2, 3, 18).

From a clinical standpoint, these findings support a risk-adapted management strategy. Current ENETS guidelines allow consideration of endoscopic resection for selected duodenal NENs measuring 5–15 mm in the absence of high-risk features and with negative endoscopic ultrasound staging. However, our results indicate that the probability of lymph-node involvement increases progressively beyond 10 mm, even within the ≤20 mm size range. Given that nodal metastases may be microscopic and not detectable by standard imaging modalities, tumors exceeding 10 mm should prompt thorough pre-treatment staging and careful patient counseling when an endoscopic approach is considered. In this setting, comprehensive staging should include endoscopic ultrasound and cross-sectional and functional imaging – preferably 68Ga-SSA-PET-CT – to minimize the risk of understaging. In this context, tumor size should be regarded as a stratification marker informing the risk of occult disease rather than as a sole determinant of treatment selection. Small (≤10 mm), low-grade, localized tumors may be adequately treated by endoscopic or limited surgical resection with close follow-up. Lesions above this threshold, or with intermediate-to-high grade, merit full staging – including lymph-node evaluation – and discussion within a multidisciplinary setting (12, 16, 19). The persistence of a protective effect of radical surgery reinforces the importance of achieving oncologic completeness in appropriately selected patients. However, this finding should be interpreted with caution, as patients undergoing radical surgery are likely to represent a selected subgroup with more favorable baseline characteristics, including younger age, better performance status, and potentially resectable disease. As such, the observed protective effect may partly reflect selection bias inherent to observational registry-based studies.

Scientifically, this analysis introduces a causal mediation framework that distinguishes the indirect effect of tumor size through stage and grade. The minimal mediated proportion demonstrates that size exerts its apparent impact almost entirely via these variables, strengthening the rationale for multidimensional prognostic models that integrate morphologic, biologic, and surgical factors. This concept aligns with the growing movement toward biology-based stratification systems in neuroendocrine neoplasms, where dimensional criteria alone are insufficient to capture clinical heterogeneity.

### Limitations

Several limitations must be acknowledged. The retrospective, registry-based design carries risks of incomplete or inaccurate coding. The SEER dataset lacks detailed information on Ki-67 index, lymph vascular or perineural invasion, and functional status and provides no centralized histopathologic review or data on recurrence, precluding evaluation of disease-free survival. Tumor size measurement in SEER may derive from different diagnostic modalities depending on clinical context, particularly in non-resected cases, and the lack of information on the source of measurement represents an inherent limitation of registry-based analyses. The SEER database does not allow reliable distinction between active surveillance and other non-surgical management strategies, nor does it provide sufficient detail to precisely classify all local or endoscopic procedures, which may be included in the ‘surgery not otherwise specified’ category. The number of lymph nodes examined varied widely, potentially introducing staging bias. Despite these constraints, the large sample size, population coverage, and DSS endpoint confer strong external validity to the findings.

## Conclusion

In duodenal neuroendocrine neoplasms measuring ≤20 mm, tumor size >10 mm is tightly linked to advanced stage and nodal involvement but does not independently influence DSS once grade, stage, and surgical treatment are considered. Tumor size, therefore, functions as a stratification marker rather than an autonomous prognostic factor.

A 10 mm cutoff effectively identifies patients at a higher risk of non-localized disease and assists in tailoring staging and surgical management. Prognosis remains governed by tumor grade, disease extent, and surgical radicality. These results provide quantitative, population-based support for the dimensional thresholds adopted in current ENETS guidelines and reinforce the need for biologically integrated prognostic models in small duodenal NETs.

## Declaration of interest

The authors declare that there is no conflict of interest that could be perceived as prejudicing the impartiality of the work reported.

## Funding

This research did not receive any specific grant from funding agencies in the public, commercial, or not-for-profit sectors.

## Author contribution statement

GL, DC, and FP conceived the study. DC curated and analyzed the dataset. GL, FZ, IZ, EA, EDU, and RDP contributed to data interpretation and manuscript drafting. DC supervised the study. All authors critically revised the manuscript and approved the final version.

## Data availability

The data supporting this study were derived from the Surveillance, Epidemiology, and End Results (SEER) program. Restrictions apply to the availability of these data, which were used under license for this study. Data are available from SEER (https://seer.cancer.gov) upon reasonable request and with permission of the National Cancer Institute.

## Declaration of generative AI in scientific writing

During the preparation of this manuscript, the authors used GPT-5.0 (OpenAI) to improve the quality of the English language and the clarity of expression. After using this tool, the authors reviewed and edited the content as needed and take full responsibility for the scientific accuracy and integrity of the manuscript.
